# Thunderstorm

**Published:** 1840

**Authors:** 


### A HAIL STORM.

On Friday, the 8th of May, about half past 5 in the afternoon, the southern part of the city of Louisville and the adjoining suburb, were visited with a thunder gust, on which we made some observations, strongly confirmative of professor Espy's theory of storms.

After a cool morning, the day became rather oppressively hot, and between four and five, P. M., a cloud was seen forming in the west. Placing ourselves at a southern chamber window, we waited its growth until it extended quite to the horizon in the east. West of our meridian the wind at the surface of the earth was to the east, and such was the course of the clouds; over our heads, the lower clouds passed rapidly to the south, while in the eastern part of the heavens, they moved to the west and south-west. Thus it could be distinctly seen that they concentrated south of us, where they soon exhibited an extensive black mass, which, reaching to the horizon on that side, prevented, no doubt, our seeing any of them coming to the common centre from that quarter. Here then were all the phenomena, which Mr. Espy's theory demands—phenomena which conclusively show, that there were horizontal currents from without inwards, and they could only have been generated by upward currents, from the spot to which they tended. These clouds, presented in the west, the peculiar dull, greenish hue, which so infallibly indicates approaching hail; and, accordingly, after a copious shower of rain, the fall of hail and the size of the hail stones, were unusually great. Now, as the general course of the cloud was from W. N. W to E. S. E., and the wind at the *surface* of the earth was chiefly in the same direction, it might have been expected, that the hail stones would have come from the same quarter, but such was not the fact, as the windows of the northern and not the western sides of our houses were broken. In the north side of the Louisville Medical Institute, upwards of one hundred and twenty panes of glass were destroyed, while none were broken on the western side. We respectfully invite our readers to direct their attention, throughout the present summer, to the course of the winds at the earth's surface and above, at the time of our thunder gusts, and thus, for themselves, decide the truth or falsehood of professor Espy's theory.

Since the above was written, intelligence has reached us that about 10 o'clock, of the day previous to this storm, the city of Natchez was visited by one of the most desolating tornadoes, ever experienced in the United States. We hope that some of our friends in that city or its vicinity, will give us their observations upon it.

